# Inhibition of hepatocelluar carcinoma MAT2A and MAT2beta gene expressions by single and dual small interfering RNA

**DOI:** 10.1186/1756-9966-27-72

**Published:** 2008-11-21

**Authors:** Qun Wang, Quan-yan Liu, Zhi-Su Liu, Qun Qian, Quan Sun, Ding-yu Pan

**Affiliations:** 1 Department of General Surgery, Zhongnan Hospital of Wuhan University, No. 169, Donghu Road, Wuhan 430071, Hubei Province, PR China

## Abstract

RNA interference (RNAi) has been successfully applied in suppression of hepatic cancer genes. In hepatocelluar carcinoma cell, one methionine adenosyltransferase (MAT) isozyme, MATII was found to have two catalytic subunits which were encoded by MAT2A and MAT2β respectively. During tumorigeness of hepatocelluar carcinoma, expressions of the two genes were discovered to be increased combining with a switch of MAT (form MATI to MATII), To figure out the role played by MATII in hepatic cancer, In this study, for the first time we established a dual small interfering RNA (siRNA) expression system, which could simultaneously express two different siRNA molecules specifically targeting two genes. To test the effectiveness of this system, we applied this approach to express simultaneously two different siRNA duplexes that specifically target MAT2A and MAT2β genes of hepatocelluar carcinoma respectively in HepG2 cell. Results indicated that dual siRNA could simultaneously inhibit the expression of MAT2A and MAT2β gene by 89.5% and 97.8% respectively, In addition, dual siRNA molecules were able to significantly suppress growth of hepatocelluar carcinoma cell in vitro as well as induce apoptosis which was involved in arrest cell cycle at the G1/S checkpoint and the expressions of p21, p27 and Bax.

## Introduction

It was demonstrated that a switch in MAT expression in liver cancer (from MAT1A to MAT2A) played an important pathogenetic role by facilitating liver cancer growth [1] The importance of MAT expression on liver phenotypephenotype was confirmed in the MAT1A knockout mouse model where replacement of MAT1A with MAT2A resulted in eventual development of HCC [2,3]MAT1A is expressed mostly in normal liver and it encodes theα_1 _subunit. MAT2A encodes a catalytic subunit (α_2_) found in a native MAT isozyme (MATII) which is associated with a catalytically inactive regulatory subunit (β) encoded by MAT2β, MAT2A predominates in hepatocelluar carcinoma and facilitates liver cancer growth. It has been proved that βsubunit was associated with cirrhosis and cancer providing a proliferative advantage in hepatoma cells through its interaction with MATIIα_2 _and down-regulation of SAMe levels[4] Recently hepatocyte growth factor (HGF) which is necessary for regeneration of hepatic cell was found to promote proliferation of hepatoma cells by up-regulating the expressions of MAT2A and MAT2β at low density[5], leptin which was demonstrated to be mitogenic in human liver cancer cell lines HepG2 was also related with increasing expressions of MAT2A and MAT2β[6]. MAT2A and MAT2β must play important roles in process of hepatocelluar carcinoma, siMAT2A and siMAT2β had been constructed respectively [6,7]. To further study their roles in hepatocelluar carcinoma, for the first time we constructed a dual small interfering RNA (siRNA) expression system which containing two siRNAs (siMAT2A and siMAT2β) simultaneously mediated by lentiviral vectors successfully, As a result growth-inhibition and apoptosis were induced by siRNA MAT2A and MAT2β. Lentiviral vector encoding antisense targeting HIV envelope sequence has been used for HIV treatment in clinical trials with no obvious side effects [8,9]. Most recently, lentiviral vector containing beta-globin gene has been approved in phaseI/II clinical trials for human beta-thalassemia and sickle cell anemia gene therapy[10]. We hope that it will be used for clinical treatment of liver cancer.

Progression of cell cycle from G1 to S phase in mammalian cell is controlled by the cyclin A, D, and E, which binds to and activates different G1 kinases (CDK4/6 and CDK2). The activation of cyclinD1/CDK4, cyclinD1/CDK6 or/and cyclin E/CDK2 complex are required for transition from G1 to S phase. The phosphorylation status of retinoblastoma tumor suppressor protein (pRb) is regulated by cyclin D1/CDK4 or cyclinD1/CDK6 complex in early G1 phase; as well as cyclin E/CDK2 complex in mid-to-late G1 stage [11]. pRb is a negative regulator of cell proliferation and a potential substrate for cyclin E/CDK2 complex at the G1-to-S phase transition of the cell cycle [12]. Hypophosphorylated pRb in G1 is active for cell-growth suppression, while its phosphorylated counterpart in S/G2/M is inactive. Both p21 and p27 inhibit the activity of the cyclin D/CDK4, cyclinE/CDK2, and of cyclin A/CDK2 complexes, whereby the phosphorylation of pRb is blocked., In addition, p21 also blocks DNA replication depending on proliferation cell nuclear antigen, resulting in G1 arrest[13]. Bax was pro-apoptosis; Bcl-xL was anti-apoptosis. Bax/Bcl-xL ratio plays important roles in the apoptosis of HepG2[14]. It has been demonstrated that induction mRNA of Bcl-xS by SAMe in HepG2 cells resulted in apoptosis but SAMe had no effects on expression of Bcl-xL[15]. Here we want to know that if apoptosis induced by siMAT2A and MAT2β was related with Bax and Bax/Bcl-xL ratio.

## Materials and methods

### Constructs and Lentivirus Production

Constructs and Lentivirus production refers to a method described previously [16,17]. MAT2A gene was cloned into the Xho I and EcoR I sites of vector pEGFP-C1 (Genechem) which was driven by CMV to yield plasmid pEGFP-C1-MAT2A. MAT2B gene was cloned into pEGFP-C1 at EcoR II and XhoI sites to generate pEGFP-C1-MAT2β. Two-pair of primers 5'-CCGCTC GAGCTATG AAC GGACAGCTCAACG-3' (sense), 5'-CCGGAATTCGAATATTTAAGCTTTTT GGGCAC-3' (antisense) or 5'-CCGCTCGAGCTATGAACGGACAGCTCAACG-3' (sense); 5'-CCGGAATTCG AATATTTAAGCTTTTTGGGCAC-3', (antisense) were used to amplify the MAT2A and MAT2β gene, respectively. The PCR products were then cloned into AgeI and EcoRII sites of pGCL-GFP to generate plasmid pGCL-GFP-MAT2A and pGCL-GFP-MAT2β, in which the MAT2A or MAT2β were fused in frame with the GFP gene and the expression of the fusion gene was driven by the CMV promoter. Four regions of the MAT2A gene and four regions of the MAT2β gene were selected as the targeted sequences of siRNA in this study. To construct single siRNA expression vector, two 64 nt primers, each containing a 19 nt or 21 nt target sequence in the sense and antisense forms from different regions of the MAT2A gene or MAT2β gene as indicated below, were systhesized:

5'-GAGAGCTATTAGAGATTGT-3' (MAT2A 1siRNA);

5'-GGATACAATCTACCACCTA-3' (MAT2A 2siRNA);

5'-GTGAGAGAGAGCTATTAGA-3' (MAT2A 3siRNA);

5'-AATATCTGGTGACTGTTGC-3' (MAT2A 4siRNA);

5'-GCAGTTCATCACATCATTCAT-3' (MAT2β1siRNA);

5'-CCTTACAGAGAGGAAGACATA-3' (MAT2β2siRNA);

5'-GCTGTGACTGTTATGTTTGAT-3' (MAT2β3siRNA);

5'-GCCTCTCAACTTAATGTGGA-3' (MAT2β4siRNA).

Sense and antisense primers were then cloned into Psc siRNA plasmid (Genechem) at EcoRI and XhoI sites after annealing according to the manufacturer's instructions. To generate the dual siRNA expression plasmid, two primers 5'-CCACGAGGCGTTCATCGAGG-3'(sense), and 5'-AAGTCTTGTAGTCAAAACCT-3'(antisense) were designed to amplify a DNA fragment containing U6 promoter and MAT2A 2siRNA expression cassette from recombinant plasmid Psc-U6-MAT2A. The PCR product was then cloned into AatII and EcoRI sites of plasmid Psc-U6-MAT2β1 to generate recombinant plasmid Psc-U6-MAT2A/β, which carries two independent siRNA expression cassettes. Correct insertions of shRNA cassettes were confirmed by restriction mapping and direct DNA sequencing. Recombinant lentiviruses vectors were produced by co-transfecting 293T cells with the lentivirus expression plasmid and packaging plasmids (pHelper 1.0 including gag/pol and pHelper 2.0 including VSVG) using Lipofectamine 2000 or calcium phosphate method [18-20]. pGCL-GFP plasmids and Psc siRNA plasmid were co-transfected in 293T cells, fluorescence-activated cell sorting analysis of GFP positive in 293T cells, then MAT2A 2siRNA, MAT2β1 siRNA plasmid was chosen as effective siRNA plasmid Infectious lentiviruses were harvested at 48 and 72 h post-transfection, centrifuged to get rid of cell debris, and then filtered through 0.22-lm cellulose acetate filters[21]. The infectious titer was determined by fluorescence-activated cell sorting analysis of GFP positive in 293T cells. The virus titers were at the range of 10^9 ^transducing units/ml medium. lentiviruses vectors containing MAT2A 2siRNA, MAT2β1 siRNA and both of them simultaneous was constructed respectively, which was designated LV-siMAT2A, LV-siMAT2β and LV-siMAT2A/2β.

### Cell culture and transfection

Human hepatocelluar cancer HepG2 cell lines (purchased from Classic Specimen Culture and Storage Centre in Wuhan University) The cell lines were grown in 5% CO_2 _saturated humidity at 37°C and cultured as monolayers in RPMI 1640 supplemented with penicillin/streptomycin, 2 mM glutamine and 10%FBS. Cells were always detached using Trypsin-EDTA. for 4–5 days and then subcultured at 1×10^5 ^cells per well into six-well tissue culture plates. After 72 h of culture, the cells were transfected with siMAT2A and2β formulated into liposomes according to the manufacturer's instructions (TransMessengerTM, Qiagen, Valencia, CA). lentiviruses concentration was 0.5μl per well, and the final volume of culture medium was 2 mL per well. Growth curve was drafted. Untreated cell was used as control.

### RT-PCR and Semi-quantitative PCR measurement

HepG2 cells were transfected with LV-siMAT2A, LV-siMAT2β and LV-siMAT2A/2β respectively. At 72 h after transfection, total RNA was extracted with TRIzol reagent according to the protocol provided by the manufacturer (Invitrogen, Carlsbad, CA, USA). Sequences of sense primer and antisense primer were seen as Table 1. Reverse transcription PCR (RT-PCR) reactions were performed as follows: cDNA was amplified for 25 cycles at 95°C for 5 min, 94°C for 1 min, 60°C for 45 s, 72°C for 1 min and 72°C for 10 min. MAT2β gene cDNA was amplified for 45 cycles at 95°C for 15 s, 94°C for 5 s, 60°C for 30 s, 72°C for 30 s and 72°C for 10 min. The PCR reaction for β-actin cDNAs was performed with 30 cycles and the reaction conditions were: denaturation at 94°C for 1 min, annealing at 53°C for 2 min, and extension at 72°C for 3 min. Semi-quantitative PCR for Bax: denaturation at 94°C for 45 s, annealing at 58°C for1 min, and extension at 72°C for 1 min. p21:95°C for30 s, 55°C for 90 s and 72°C for 120 s using 30 cycles, p27:94°C 30 s, 55°C 30 s, or 57°C 30 s, 72°C 60 s. PCR products were analyzed through 1% agarose gel electrophoresis and following ethidium bromide staining.

**Table 1:** Sequences of sense primer and antisense primer

β-actin mRNA (300 bp)		
β-actin	sense primer	5'-CTGGGGCGCCCCAGGCACCA-3'
	antisense primer	5'-CTCCTTAATGTCACGCACG ATTTC-3'
MAT2A mRNA (287 bp)		
MAT2A	sense primer	5'-CCACGAGGCGTTCATCGAGG-3'
	antisense primer	5'-AA GTCTT GTAGTC AAAACCT-3'
MAT2β mRNA (324 bp)		
MAT2β	sense primer	5'-ACAGAG AGGAAGACATACCAG-3'
	antisense primer	5'-GTTCATTGCCAGACCAGTG-3'
Bax mRNA		
Bax	sense primer	5'-ACCAAGAAGCTGAGCGAGTGTC-3'
	antisense primer	5'-TGTCCA GCCCATGATGGTTC-3'[22]
p21 mRNA		
p21	sense primer	5'-AAGACCATGTGGACCTGTCA-3'
	antisense primer	5'-AGGAAGTAGCTGGCATGAAG-3' [23]
p27 mRNA		
p27	sense primer	5'-AAG TGGCATGTTTTGTGCATTT-3'
	antisense primer	5'-GCT CAG TATGCAACC TTTTAAGCA-3' [24]

### Western-blot analysis

Cells were collected after different treatments, rinsed in ice-cold PBS, 15 mins, and lysed in lysis buffer containing 50 mmol/L HEPES (pH 7.9), 0.4 mol/L NaCl, 1 mmol/L EDTA, 2 Ag/mL leupeptin, 2 Ag/mL aprotinin, 5 Ag/mL benzamidine, 0.5 mmol/L phenylmethylsulfonylfluoride, and 1% NP40. The lysates were centrifuged at 14,000 rpm to remove any cellular debris. Protein concentrations of the lysates were determined by the Bio-Rad Dc protein assay system which was 2 μg/μl. Equal amount of protein was separated by12% SDS-PAGE, transferred to PVDF membrane, and blocked with 5% nonfat dry milk in TBS/Tween 20 (0.05%, v/v) for 1 hour at room temperature. The membrane was incubated with primary antibody overnight. anti-GAPDH anti-p21, anti-p27 and anti-Bax antibodies were obtained from Santa Cruz Biotech. Anti-MAT2A antibody were obtained from Genway company. Anti-MAT2β was purchased from Abnova corporation (Tai wan, China). After washing, the membrane was incubated with secondary antibody (diluted 1:4,000; Amersham Pharmacia Biotech, Arlington Heights, IL) for 1 hour. Following several washes, the blots were developed by enhanced chemiluminescence. (Amersham, Arlington Heights, IL). Each experiment was repeated at least twice with similar results.

### Cells apoptosis analysis by Flow cytometry

The inducing apoptosis effects of LV-siMAT2A, LV-siMAT2β and LV-siMAT2A/2β on human hepatocelluar cancer cells and were analyzed by Flow cytometry. Briefly, Cells were incubated with LV-siMAT2A, LV-siMAT2β, LV-siMAT2A/2β and vehicle alone for 72 h, fixed with 70% ethanol treated with RNase (100 lg/ml, Sigma Chemical Co., St. Louis, MO), and stained with propidiumiodide (100 lg/ml, Sigma) for 30 min on ice. DNA content in the cells was analyzed by a flow cytometer (Epics XL; Beckman Coulter, Miami, FL).

### Assay of Hepatic SAMe levels

Hepatic SAMe levels were measured using a method described previously[25] with slight modifications. Liver specimens were homogenized in phosphate-buffered saline, and an aliquot was saved for protein assay. The rest was treated with 100 μl of 1 M perchloric acid (PCA) onice for 5 min and centrifuged at 1000 g for 15 min at 4°C. The aqueous layer was quantitatively removed, neutralized with 3 M KOH, and centrifuged at 3000 g for 10 min at 4°C. SAMe levels were deter mined in the neutralized PCA extracts by HPLC (LC-10ATVP pump, SCL-10AVP system control) with a SPD-10AVP UV detector and a SIL-10ADVPautosampler (Shimadzu) using a Partisil SCX 10 μm column (25 × 0.44 cm i.d.; Whatman Chem. Sep. Maidstone, Cleveland, OH). SAMe was eluted isocratically at 1 ml/min with 0.19 MNH4H2PO4 adjusted to pH 2.6 with 2 M H3PO4. SAMe levels were calculated using standard curve of SAMe prepared at the same time as the samples and are reported as nmol/mg protein.

### Statistical methods

All results are expressed as mean ± SD, and statistical analyses of the data is performed using ANOVA Post Hoc Dunnett t test. All p values are based on two-sided hypothesis testing, P < 0.05 is considered significant.

## Results

### Suppression of cell growth caused by LV-siMAT2A, LV-siMAT2β and LV-siMAT2A/2β

**Figure 1:**
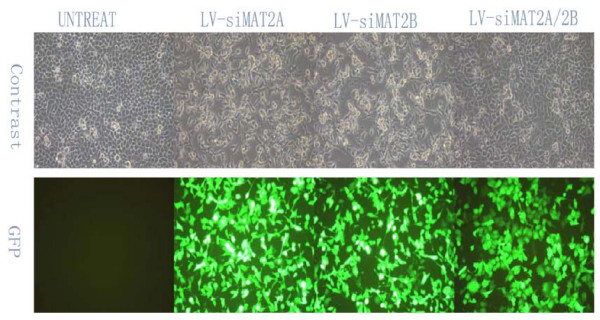
**shows that HepG2 cells were without treatment (untreat), and infected with LV-siMAT2A, ALV-siMAT2β and LV-siMAT2A/2β**. The cells were infected (MOI = 25), GFP expression and the phase contrast images of same areas were taken after 72 h.

**Figure 2:**
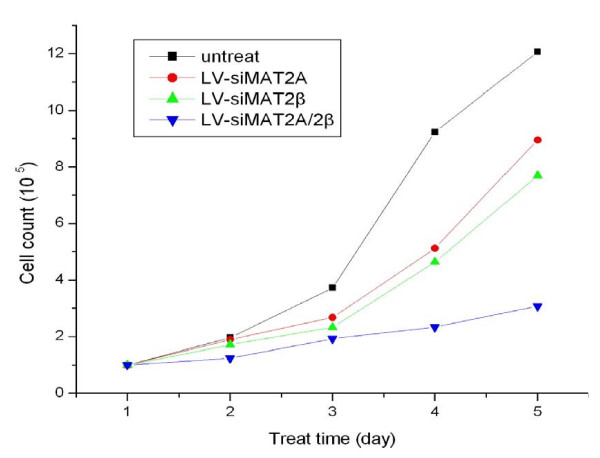
**indicates that HepG2 cells were without treatment (untreat), and infected with LV-siMAT2A, ALV-siMAT2β and LV-siMAT2A/2β, in the following 5 days cells were counted every day, then growth curve was drafted**.

To assess the potential effects of Lentivirus vectors RNAi-mediated MAT2A and MAT2β silencing on cell growth, LV-siMAT2A, LV-siMAT2β and LV-siMAT2A/2β were transfected into respectively, 72 hours after infection at multiplicity of infection of 25 through estimating GFP expression under a fluorescent microscope (Figure 1). Cells were counted every day for 5 days after transfections, growth curve was drafted. As a result, growth-inhibition effects were produced by LV-siMAT2A, LV-siMAT2β and LV-siMAT2A/2β, in which caused by LV-siMAT2A/2β was most evident (p < 0.01) and LV-siMAT2A was most slight(p < 0.05) (Figure 2)

### Knockdowns of MAT2A, MAT2β and both by LV-siMAT2A, LV-siMAT2β and LV-siMAT2A/2β respectively could Induce Apoptosis in human hepatocelluar cancer cell

**Figure 3:**
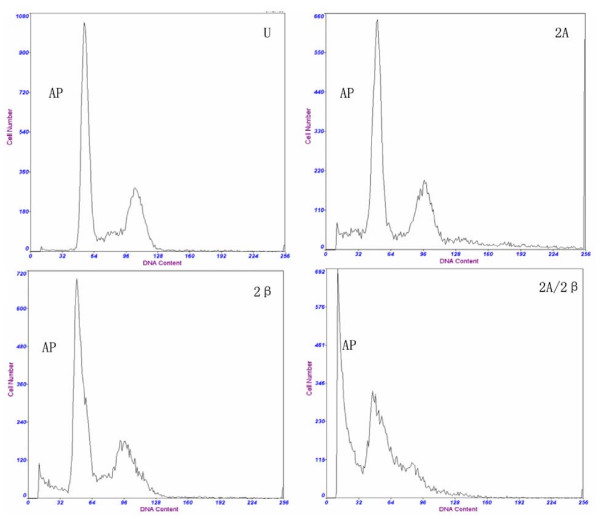
**shows that HepG2 cells without treatment (U), and were infected with LV-siMAT2A(2A), ALV-siMAT2β (2β)and LV-siMAT2A/2β (2A/2β), cell apoptosis was induced by LV-siMAT2A, ALV-siMAT2β and LV-siMAT2A/2β, as showed in the picture "AP" stands for apoptotic peak, There was most cells into apoptosis after cells were deal with LV-siMAT2A/2β, and G1/S arrest was discovered**. It was demonstrated in the Fig. 3 that cell apoptosis caused by LV-siMAT2A/2β was increased in human hepatocelluar cancer cells HepG2 compared to that induced by LV-siMAT2A, ALV-siMAT2β (P < 0.01) and control, but they all had no difference between LV-siMAT2A and LV-siMAT2β.

**Figure 4:**
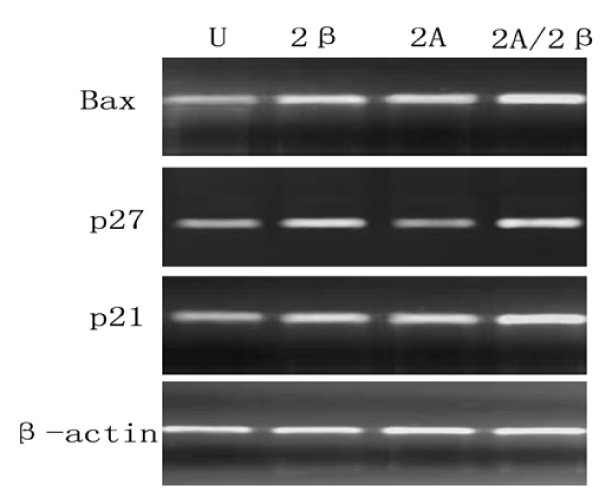
**displayed that HepG2 cells without treatment (U), and were infected withLV-siMAT2β (2β), A LV-siMAT2A(2A)and LV-siMAT2A/2β (2A/2β)**. PCR was applied to assay the expressions of Bax, p27 and p21 β-actin was used as control, It was showed in Fig 7 that mRNA of Bax and p21 was increased by LV-siMAT2β, ALV-siMAT2A and LV-siMAT2A/2β, which were both highest after treating with LV-siMAT2A/2β, mRNA of p27 was increased by LV-siMAT2β and LV-siMAT2A/2β but not by LV-siMAT2A

**Figure 5:**
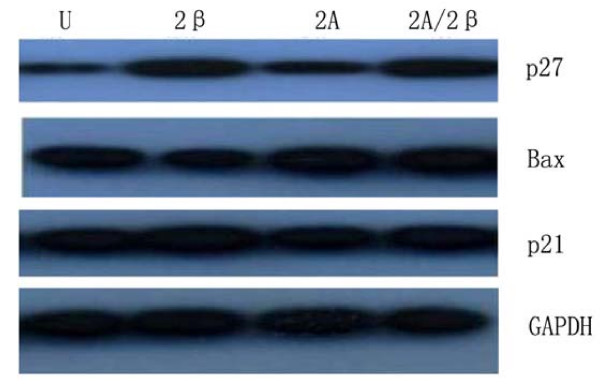
**showed that HepG2 cells without treatment (U), and were infected withLV-siMAT2β (2β), A LV-siMAT2A(2A)and LV-siMAT2A/2β (2A/2β), LV-siMAT2β, ALV-siMAT2A and LV-siMAT2A/2β**. Western-blot was applied to assay the expressions of Bax, p27 and p21 GAPDH was used as control, It was showed in Fig 8 that protein of Bax was increased by LV-siMAT2β, ALV-siMAT2A and LV-siMAT2A/2β, which was highest after treating with LV-siMAT2A/2β, protein of p27 was increased by LV-siMAT2β and LV-siMAT2A/2β but not by LV-siMAT2A, The expression of p21 does not change.

To investigate the effects of LV-siMAT2A, LV-siMAT2β and LV-siMAT2A/2β on cell apoptosis. The distribution of cell cycle was determined by flow cytometry analysis. After HepG2 cells were transfected with LV-siMAT2A, LV-siMAT2β and LV-siMAT2A/2β for 72 h, the percentage of both S and G2/M phase were decreased 72 h after LV-siMAT2A, LV-siMAT2β and LV-siMAT2A/2β treatment, and the percentage of G0/G1 phase was increased, (Figure. 3). The expressions of p21 and Bax were up-regulated by LV-siMAT2A, LV-siMAT2β and LV-siMAT2A/2β. p27 which was up-regulated by LV-siMAT2β and LV-siMAT2A/2β but not by LV-siMAT2A. the mRNA levels of p21 and Bax were highest induced by LV-siMAT2A/2β, but that there was no difference between that caused by caused LV-siMAT2A LV-siMAT2β (Figure 4). Western-blot was applied to assay the expressions of Bax, p27 and p21, After HepG2 cells were transfected with LV-siMAT2A, LV-siMAT2β and LV-siMAT2A/2β for 72 h, GAPDH was used as control, It was showed in figure 5 that protein of Bax was increased by LV-siMAT2β, LV-siMAT2Aand LV-siMAT2A/2β, which was highest after cells was deal with LV-siMAT2A/2β, protein of p27 was increased by LV-siMAT2β and LV-siMAT2A/2β but not by LV-siMAT2A, The protein of p21 does not change compared with control.

### Lentivirus-Mediated RNAi Efficiently Suppressed MAT2A and MAT2β mRNA in HCC HepG2 cell in vitro

**Figure 6:**
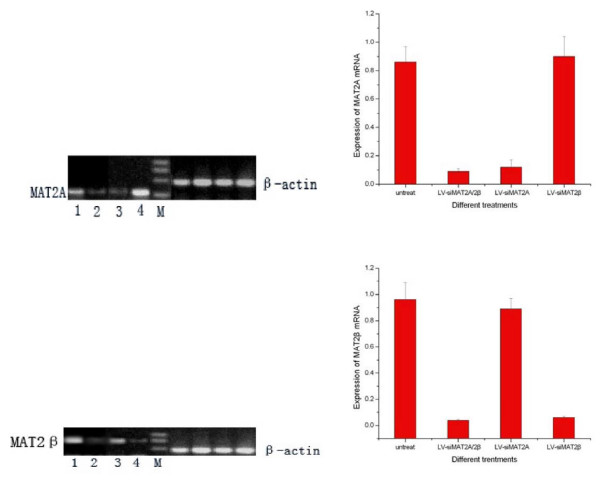
**demonstrated that after HepG2 cells were done with different treatment**. RT-PCR was performed to decide the mRNA level of MAT2A and MAT2β, 1 means untreated cells; 2, 3 and 4 stands for doing withLV-siMAT2A/2β, LV-siMAT2A and LV-siMAT2β respectively. It was showed in the Fig 4 that MAT2A and MAT2β were suppressed byLV-siMAT2A and LV-siMAT2β respectively and by LV-siMAT2A/2β simultaneously. LV-siMAT2A has on effect on expression of MAT2β, vice versa.

After HepG2 cells were transfected with LV-siMAT2A, LV-siMAT2β and LV-siMAT2A/2β for 72 h, RT-PCR was performed to determined the mRNA of MAT2A and MAT2β. As it showed in figure 6 mRNA of MAT2A and MAT2β were knocked down by LV-siMAT2A, LV-siMAT2β by 85.5% and 94.0% respectively, and both of them were suppressed LV-siMAT2A/2β by 89.5% and 97.8% simultaneously compared with control which was untreated. We found that LV-siMAT2A had no effect on mRNA of MAT2β, on the other hand, LV-siMAT2β had no impact on mRNA of MAT2A.

### Lentivirus-Mediated RNAi Efficiently Suppressed MAT2A and MAT2β protein in HCC HepG2 cell in vitro

**Figure 7:**
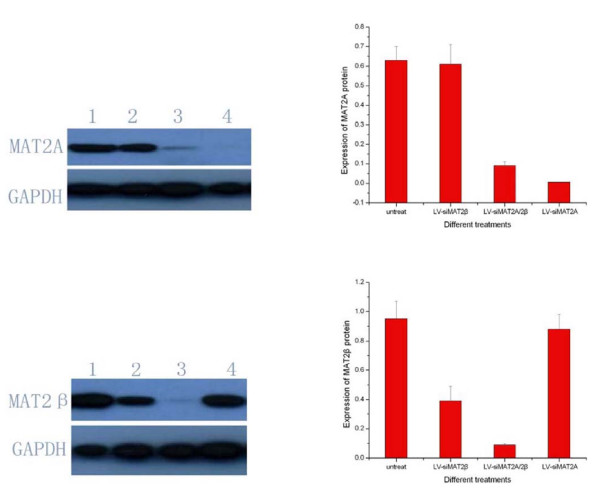
**showed that after HepG2 cells were done with different treatment**. Western-blot was performed to detect the protein level of MAT2A and MAT2β, 1 means untreated cells;2,3 and4 represents for doing withLV-siMAT2β, LV-siMAT2A/2β and LV-siMAT2A respectively. It was showed in the Fig 5 that MAT2A and MAT2β were suppressed byLV-siMAT2A and LV-siMAT2β respectively and by LV-siMAT2A/2β simultaneously.

In order to figure out the suppression efficiencies caused by LV-siMAT2A, LV-siMAT2β and LV-siMAT2A/2β, Western-blot was applied to detect protein of MAT2A and MAT2β after HepG2 cells were transfected with LV-siMAT2A, LV-siMAT2β and LV-siMAT2A/2β for 72 h. It was indicated in figure 7 that the expressions of MAT2A and MAT2β were inhibited by LV-siMAT2A and LV-siMAT2β respectively at protein level by 99.4% and 60.2%, both of them were suppressed by LV-siMAT2A/2β by 98.6% and 90.8%. We discovered that LV-siMAT2A did not have impact on protein expression of MAT2β, and LV-siMAT2β also did not have effect on impact on protein expression of MAT2A too.

### Intracellular SAMe Contents were changed by LV-siMAT2A, LV-siMAT2β and LV-siMAT2A/2β

**Figure 8:**
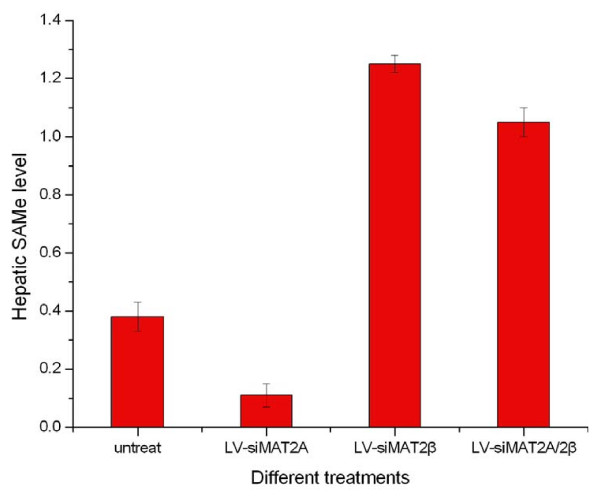
Indicated that after HepG2 cells were transfected LV-siMAT2A, ALV-siMAT2β and LV-siMAT2A/2β respectively, compared with control Hepatic SAMe level was decreased by LV-siMAT2A (P < 0.01), and increased by LV-siMAT2β and LV-siMAT2A/2β (P < 0.01), but there was no difference between LV-siMAT2β and LV-siMAT2A/2β.

The changes in MAT genes expression are likely to cause changes in the production of SAMe. We analyzed the effects of silencing MAT2A and MAT2β by different Lentivirus-Mediated RNAi on SAMe production by measuring levels of SAMe a in HCC cell. As a result, Intracellular SAMe content was decreased by LV-siMAT2A and increased by LV-siMAT2β as well as LV-siMAT2A/2β, compared with control. (Figure 8)

## Discussion

Methionine adenosyltransferase enzymes (MAT I, MAT II, and MAT III) have been demonstrated to play roles in hepatic cirrhosis and cancer. [26,27] Two different genes, MAT1A and MAT2A, encode for two homologous MAT catalytic subunits, α_1 _andα_2_. [28-30] MAT1A is expressed mostly in the normal adult liver, and it encodes theα_1 _subunit discovered in two native MAT isoenzymes, which are either a dimer (MAT III) or tetramer (MAT I) of this single subunit.[30] The gene MAT2A encodes for the α_2 _catalytic subunit in the MATII isoform. The gene MAT2β, encodes for a β regulatory subunit that regulates the activity of MATII by lowering inhibition constant (Ki) for SAMe and Michaeli's constant (Km) for methionine [1], MAT2A and its gene product also predominate in the fetal liver and are progressively replaced by MAT1A during development in normal adult liver [31,32] but in liver cirrhosis and cancer, expressions of MAT2A and MAT2β are both increased. MAT1A gene is specifically silenced by hypermethylation in human cirrhosis, which leads to a marked reduction of SAMe synthesis, We discovered the SAMe content could be decreased by knocking down MAT2A and increased by knocking down MAT2β which were in agreement with the results of Komal[6]. And we further discovered that the level of SAMe was increased when MAT2A and MAT2β were suppressed by LV-siMAT2A/2β simultaneously. This may be related with decreased expression of MAT2β which was believed to regulate MATII activity by increasing the sensitivity of the enzyme to feedback inhibition by SAMe[33]. It has been demonstrated that the type of MAT expressed by the cell significantly influenced the rate of cell growth. Further research discovered that expression of MAT2A not only correlated with liver cancer cell proliferation but also was necessary for this process [34]. The β subunit was associated with cirrhosis and cancer providing a proliferative advantage in hepatoma cells through its interaction with MAT II α_2 _and down-regulation of SAMe levels[4]. That is to say that both MAT2A and MAT2β can facilitate liver cancer growth and it was interested to find that when MAT2A and MAT2β were suppressed by LV-siMAT2A and LV-siMAT2β respectively, apoptosis and growth-inhibition were both induced, LV-siMAT2A had no effect on expression of MAT2β and LV-siMAT2β had no effect on expression of MAT2A at either mRNA or protein levels. We hypothesized that MAT2A and MAT2β may influence growth of liver cancer independently, this is the reason why we want to construct the dual small interfering RNA expression system targeting to MAT2A and MAT2β simultaneously. As a result we came to find that the effect of growth-inhibition and apoptosis caused by LV-siMAT2A/2β was more than that caused by LV-siMAT2A and LV-siMAT2β, The changes in MAT genes expression are likely to cause changes in the production of SAMe. SAMe plays an important role in growth and apoptosis of hepatic cell [35,36] But it was showed in our research that both LV-siMAT2A and LV-siMAT2β, as well as LV-siMAT2A/2β can induce growth-inhibition and apoptosis in hepatic cancer HepG2 cell in spite of level of SAMe. We, therefore, considered that besides regulating intracellular SAMe content there must be another pathway for MAT2A and MAT2β to make impact on cell growth and apoptosis. As a result we revealed that the expression of p21 that inhibited G1 to S phase transition was up-regulated and Bax which was regarded as pro-apoptotic gene [37] was increased by LV-siMAT2A LV-siMAT2β and LV-siMAT2A/2β, the mRNA levels of p21 and Bax were highest induced by LV-siMAT2A/2β, but that there was no difference between that caused by caused by LV-siMAT2A and LV-siMAT2β. The expression of Bax may be related with that of MAT2A and MAT2β, because Leptin was demonstrated to be mitogenic in human liver cancer cell lines HepG2 which was associated with increasing of MAT2A and MAT2β simultaneously[6] and Leptin has been proved to induce proliferation and anti-apoptosis in human hepatocarcinoma cells by up-regulating cyclin D1 and down-regulating Bax via a Janus kinase 2-linked pathway[38]. Although had no effects on expression of Bcl-xL, but expression of Bax was increased, Bax/Bcl-xL ratio was changed so apoptosis was induced. On the other hand, it was demonstrated by us that p27 which was believed to be cell-cycle regulators at S phase cell-cycle arrest [39-42] was up-regulated by LV-siMAT2β and LV-siMAT2A/2β but not by LV-siMAT2A, To agree with this that the percentage of S phase in cell treated with LV-siMAT2A was more than that caused by LV-siMAT2β and LV-siMAT2A/2β and grow-inhibition effect was more evident in hepatic cancer HepG2 cell transfected with LV-siMAT2β and LV-siMAT2A/2β. We further applied western-blot to assay the proteins of Bax, p21 and p27, we found that protein of Bax was increased by LV-siMAT2A, LV-siMAT2β and LV-siMAT2A/2β, that of p27 was up-regulated by LV-siMAT2β and LV-siMAT2A/2β but not by LV-siMAT2A, the expression of p21 was not changed by all of them at protein level, this may because p21 is regulated mainly at transcriptional level by p53-dependent or independent mechanisms[43]. Whereas p27 is regulated predominantly at post-translational level, especially protein degradation[44]. We also came to a conclusion that the expression of p27 was related with MAT2β but not MAT2A and the expression of p21 was regulated by both MAT2A and MAT2β.

In total, we had a conclusion that the dual small interfering RNA expression system targeting to MAT2A and MAT2β simultaneously was constructed successfully, LV-siMAT2A/2β can lead to grow-inhibition and cell apoptosis which is more than LV-siMAT2A and LV-siMAT2β, this may be a new way for the therapy of hepatic cancer in the future.

## Competing interests

The authors declare that they have no competing interests.

## Authors' contributions

QW and LZS contributed equally to this work. QW, QYL and ZSL designed research. QW, QYL performed research. QQ and QS contributed new reagents/analytic tools. QW, QYL, ZSL and DYP analyzed data. QW wrote the paper.
